# Physical and Chemical Properties of Rat Taro Simplicia From Various Drying Methods

**DOI:** 10.1155/tswj/6239186

**Published:** 2025-12-05

**Authors:** Bagem Br Sembiring, Sintha Suhirman, Christina Winarti, Helmi Haris, Tika Hafzara Siregar, Erma Maryana, Niken Harimurti, Feri Manoi, Iceu Agustinisari

**Affiliations:** ^1^ Research Center for Equipment Manufacturing Technology, National Research and Innovation Agency, Tangerang, Indonesia, brin.go.id; ^2^ Research Center for Process Technology, National Research and Innovation Agency, Tangerang, Indonesia, brin.go.id; ^3^ Research Center for Food Technology and Processing, National Research and Innovation Agency, Yogyakarta, Indonesia, brin.go.id; ^4^ Food Technology Department, University of Djuanda, Bogor, West Java, Indonesia; ^5^ Directorate for Laboratory Management, Research Facilities, and Science and Technology Park, National Research and Innovation Agency, Tangerang, Indonesia, brin.go.id; ^6^ Research center for sustainable industrial and manufacturing systems, National Research and Innovation Agency, Tangerang, Indonesia, brin.go.id

**Keywords:** antioxidant activity, drying, flavonoid, proximate, *Typhonium flagelliforme*

## Abstract

Rat taro is a potential plant as a raw material for medicine. Rat taro contains an active compound that is useful as an anticancer, antibacterial, and antioxidant. Standardization of raw materials is important to obtain reproducible effects and safe consumption. This study is aimed at obtaining an appropriate drying method to produce high‐quality rat taro simplicia from the tuber and leaf. The drying methods applied were sun drying, modified sun drying covered with black fabric, and oven drying. Simplicia of rat taro was made from the tuber and leaf. Simplicia quality was observed for moisture content, dry weight yield, ash content, water‐ and alcohol‐soluble essence, flavonoids, and antioxidant activity (IC_50_). The results showed that the average drying temperature for the tuber was around 37.5°C–39°C, and for the leaf, it was 32.5°C–37°C. The drying time for the tuber was around 15–16 h, and for the leaf, it was 20–26 h. The average moisture content of rat taro simplicia ranged from 8.27% to 10.84%. The water‐soluble extract content of the tuber was around 10.92%–12.81%, and of the leaf, it was 35%–39.10%. Alcohol‐soluble extract content of the tuber was 1.88%–2.28%, and of the leaf, it was 6.35%–9.19%. The flavonoid content of the tuber was around 0.2–0.3 mg QE/g, and of the leaf, it was 4.1–5.6 mg QE/g. The IC_50_ value of the tuber was around 6477.70–8847.77 ppm, and of the leaf, it was 585.01–1189.63 ppm. The results revealed that the drying method influenced antioxidant activity (*p* = 0.028) and flavonoid levels (*p* = 0.009) in rat taro simplicia. The moisture content, antioxidant activity, and flavonoid content of the leaves were higher than the tubers. The most effective drying method for rat taro simplicia was oven drying at 37°C–38°C, which yielded flavonoid contents of 0.3 mg QE/g in the stems and 5.6 mg QE/g in the leaves, along with antioxidant activities of 6477.70 ppm in the stems and 585.01 ppm in the leaves, representing the best results obtained.

## 1. Introduction

Indonesia has the second‐largest biodiversity in the world in terms of the diversity of flora and fauna [1]. Biodiversity is an important factor in the development of traditional medicine [2]. The medicinal plants have various benefits, including enhancing immune system control, inducing apoptosis, and inhibiting tumor proliferation, thereby reducing pain and sickness, increasing body resistance, and potentially aiding in the repair of damaged organs and killing disease germs [3]. Rat taro (*Typhonium flagelliforme*) bioactive compounds exhibit various pharmacological effects, including anticancer activities against human lung, breast, prostate, and colon cancer cells, as well as anti‐inflammatory, antioxidant, antiviral, antiallergic, neuroprotective, and hepatoprotective properties. The nutritious compounds in rat taro plants include amino acids, fatty acids, flavonoids, and vitamins [4]. Rat taro plant extract also has the potential as a fungicide, antibacterial, and antioxidant [5]. Flavonoids stimulate intestinal absorption to be more stable and can inhibit the growth of pathogenic bacteria in the large intestine [6]. The active compounds of rat taro that have anticancer potential are hexadecenoic acid, stearic acid, and oleic acid [7].

Simplicia is a form of product used as a raw material for herbal medicine. The most important step to anticipate is maximizing the standardization of raw materials to obtain reproducible effects and safe consumption. Herbal products in the form of simplicia are produced through a drying process of fresh ingredients. The speed of the drying process is related to moisture content and temperature. The lower the water content and the higher the temperature, the faster the drying process. Plant bioactive compounds are sensitive to heat. Drying at high temperatures can damage or degrade the active compounds contained in the material [8]. The previous study examines the detrimental effects of hot air drying on phenolic and flavonoid contents, where the presence of oxygen during drying leads to significant losses [9]. Furthermore, the increased drying temperatures can result in the synthesis of new phytochemicals. Elevated drying temperatures result in the synthesis or increase the compounds such as 2,3‐dihydrobenzofuran, tricyclopentadeca‐3,7‐dien, and 2,3‐dihydro‐3,5‐dihydroxy‐6‐methyl‐4H‐pyran‐4‐one. This modification is probably attributable to oxidative processes and cell wall breakdown, underscoring how drying can result in both losses and the formation of new molecules [10]. The drying affects the concentrations of essential bioactive compounds in bulgur [11]. The quality of simplicia is influenced by water content and bioactive compound content. Moreover, the color and aroma should not be far from the original.

Drying can decrease the quality of the material and also can reduce the weight and volume of the product [12]. Drying technique affects the physical properties and reconstitution behavior of taro flour [13]. The previous study revealed that exposure to high temperatures during processing may have significantly influenced the decrease in nutritional and phytochemical levels, as well as the antinutrient content of raw taro [14]. Another research found that drying conditions are crucial for maintaining functional quality and nutrient density of taro [15]. They specifically noted that improper drying conditions could lead to degradation of nutritional content and functionality. Drying can stop enzymatic reactions and prevent material quality degradation. Apart from drying air conditions, the type and composition of agricultural materials are also related to the drying rate [16]. Therefore, the drying process must be carried out under the right conditions [17]. Previous studies reported that drying rat taro leaves using an electric oven at 40°C and 50°C was inefficient, as the process required more than 20 h to reduce the moisture content below 10% [18]. Moreover, the effect of drying temperature on the chemical properties of rat taro simplicia has not yet been elucidated. The effect of drying methods on the physical and chemical properties of rat taro simplicia has not been extensively reported.

Therefore, this study explores a more efficient drying method that shortens drying time while comparing the effect of physical and chemical quality of rat taro simultaneously. This research is aimed at obtaining a drying method to produce good‐quality simplicia of rat taro leaves and tubers to meet efficiency and efficacy in the production process. This research can support small‐scale industries to produce raw materials in high quantity, quality, and continuity to meet the standardized herbal medicines.

## 2. Materials and Methods

### 2.1. Materials

**Figure 1:**
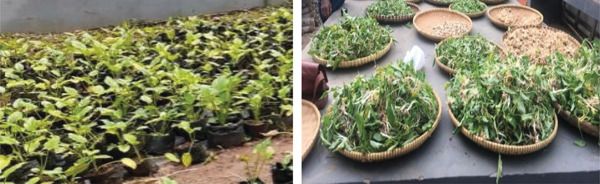
Postharvest handling and drying of rat taro.

Eight‐month‐old tubers and leaves of rat taro were obtained from the Spice and Medicinal Plants Research Institute, Bogor, Indonesia. Rat taro was cleaned of dirt; sorted from weeds, soil, and yellow leaves; and washed under tap water until completely clean. Leaves and tubers were chopped separately. The chopping technique is carried out transversely with a chopping thickness of around 3 mm for tubers and 3–4 mm for leaves using a clean and sharp knife made of stainless steel. Figure 1 shows postharvest handling and drying of rat taro. Tuber and leaves were separated, and each part was weighed. Chemicals consisting of distilled water, 2,2‐diphenyl‐1‐picrylhydrazyl (DPPH) (TCI, Japan), methanol (Merck), and Whatman Filter Paper No. 41 were used for extraction.

### 2.2. Drying Experiments

Tubers and leaves of rat taro were separately dried using various methods. The drying method consisted of three drying methods with three repetitions. Each type of dryer is equipped with a data logger (Graphtec Midi Logger GL240) to record temperature and humidity during the drying process. The drying method used to dry the rat taro was sun drying (traditional drying), modified sun drying with black fabric as a cover, and oven drying. The sun dryer consisted of a tray or rack made of porous wire (SS 304 mesh 16). The rat taro tuber and leaf were sliced and placed in the sunlight. The modified sun dryer was a drying method under the sunlight using a tray made of porous wire (SS 304 mesh 16) and covered with black fabric. The oven dryer (Hinode, Japan) was square‐shaped with a single rack positioned at the center, on which the samples were placed. Temperature and humidity sensors (DHT22) were installed above the samples and connected to a data logger. A natural gas‐powered heater combined with an electric blower was located beneath the rack to deliver hot air for drying the samples. The airflow velocity was 0.495 ± 0.09 m/s, and the oven temperature was maintained manually at a maximum of 50°C. The materials are weighed and placed in the drying rack. Figure 2 shows three drying methods.

Figure 2Drying methods: (a) sun drying, (b) modified sun drying, and (c) oven drying.

**Figure:**
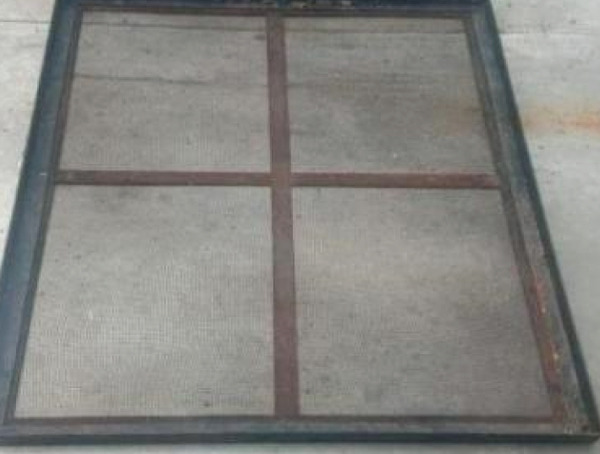
(a)

**Figure:**
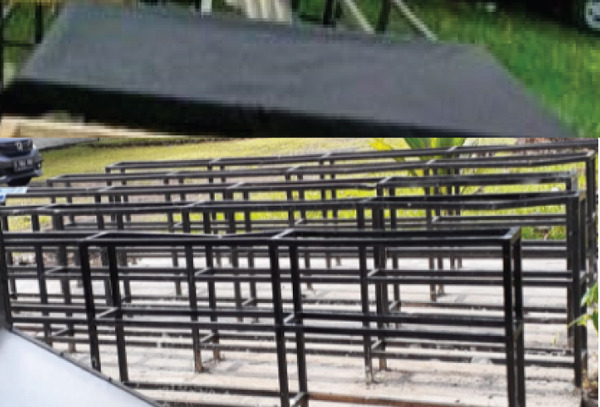
(b)

**Figure:**
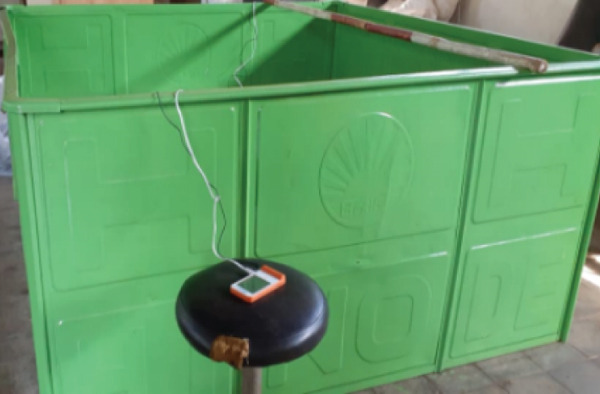
(c)

### 2.3. Observation Parameters

The observed parameters to produce the simplicia are temperature and relative humidity (RH) for each drying method. Simplicia quality was observed for moisture content, dry weight yield, ash content, water‐ and alcohol‐soluble extract content, flavonoids, and antioxidant activity (inhibition concentration 50% (IC_50_)). Moisture content was analyzed with the distillation method using toluene (SNI 01‐2891:1992). For ash content measurement, weigh 3 g of fine sample, add it to a silicate crucible that has been ignited and tarred, ignite slowly until the charcoal is finished, allow it to cool, and then weigh. The total ash content is calculated from the weight of the test material, expressed in percentage *w*/*w*. Water‐ and alcohol‐soluble extract content was analyzed by the gravimetric method from guidelines described in Indonesian Herbal Pharmacopoeia, 2nd Edition, 2017. The measurements were carried out in three repetitions.

### 2.4. Flavonoid Content

Rat taro simplicia, consisting of tubers and leaves, was weighed 0.1 g each and then dissolved in 100 mL of 96% proanalysis ethanol (Merck). Pipette 2 *μ*L of the extract solution with a micropipette, and then, spot on a cellulose TLC plate with a size of 10 × 10 cm and an elution distance of 7 cm. The TLC plate was eluted with the mobile phase of acetic acid and water (50:50). Then, the plate was tested by densitometry at a maximum wavelength of 327 nm. The total flavonoid content in rat taro simplicia extract was calculated using a standard curve of quercetin. The determination of the maximum wavelength (*λ* max) for quercetin was conducted by scanning a quercetin solution within the UV–Vis using an ELISA Reader (BioTek Synergy HTX Multi‐Mode). Quercetin (Merck) was used as the standard at concentrations of 2, 4, 6, 8, 10, and 12 ppm. The flavonoid content of the extract was expressed in quercetin equivalents (mg QE/g extract).

### 2.5. Antioxidant Activity Test

The antioxidant activity of rat taro extract was tested using the DPPH method [19]. The extract solutions were prepared by pipetting 0.5, 1.0, 1.5, 2.0, and 2.5 mL of the stock solution, each diluted with methanol (99.98%, Merck) and water (1:1) in a 10‐mL volumetric flask to the mark, yielding final concentrations of 50, 100, 150, 200, and 250 *μ*g/mL. From each concentration, 1 mL of the extract solution was transferred into a test tube, followed by the addition of 1 mL of DPPH solution (100 *μ*g/mL, TCI, Japan) and 2 mL of methanol. The mixture was homogenized and then left for 30 min in a dark place. The absorbance (*A*) of the solution was measured using a UV–Vis spectrophotometer (Hitachi U‐2010) at a wavelength of 515.5 nm with ascorbic acid 2 ppm as a reference. The effectiveness of antioxidants is determined by the IC_50_ expressed in parts per million. The IC_50_ value shows that the number of extracts tested can capture 50% of free radicals, as shown by the linear regression line equation which states the relationship between the concentration of the sample compound (*X*) and the average antioxidant activity/inhibition (*Y*) which is a value that shows the amount of extract tested that can capture 50% of free radicals.

(1) Inhibition concentration IC50=A control−A sampleA sample×100%.

### 2.6. Statistical Analysis

The activity used a completely randomized design consisting of two factors. The first factor, namely, plant parts, was tubers and leaves. The second factor was the drying method, which consisted of three drying methods with three repetitions. Analysis of variance (ANOVA) was used to identify the significance of treatment condition on each response, and Duncan′s multiple range test was used for mean comparison at the 0.05 significance level. All statistical analyses were performed using SPSS for Windows Version 18.0 (SPSS Inc., Chicago, Illinois, United States).

## 3. Result and Discussion

### 3.1. Drying Condition

#### 3.1.1. Sun Drying Method

Drying under sunlight is one of the most traditional methods employed to decrease the moisture content of materials, thus enhancing their storability. In the case of rat taro (*Typhonium flagelliforme*), the drying process to obtain simplicia from the tubers requires approximately 15 h, while the leaves require about 21 h. During tuber drying, the average temperature and RH were 37.5°C and 53%, respectively, whereas for leaves, the average temperature and RH were 37.5°C and 52.5%, respectively.

#### 3.1.2. Modified Sun Drying Method

Modified sun drying takes a longer time to produce rat taro simplicia. The drying time for tubers is around 16 h and leaves 26 h. The average temperature and humidity in the modified sun dryer for tubers were 39°C and 54.5%, while the leaves′ temperature and humidity were 36°C and 46.5%.

#### 3.1.3. Oven Drying Method

The drying time for rat taro tubers is around 15 h and leaves 20 h. The average temperature and humidity of the dryer during the process were 38°C and 48% for tubers and 37°C and 51% for leaves, respectively.

### 3.2. Temperature and Humidity

Temperature and humidity can affect the speed of the drying process. To evaporate moisture from the material, drying is carried out using three different drying methods. Drying rat taro tubers into simplicia form takes around 15–16 h to reach a maximum moisture content of 10%. The average temperature during the drying process ranges from 37.5°C to 39°C and humidity 48.0% to 54.5%. Rat taro leaves took about 20–26 h to dry at a temperature of 36°C–37.5°C and a humidity of 46.5%–52.5% (Table 1). In the drying process, air temperature and humidity are inversely proportional. The higher the drying room temperature, the lower the air humidity (RH). Drying temperature can affect the quality of simplicia products. Products exposed to high drying temperatures with low RH dry faster than the products in the other drying zones [15]. The temperature of all drying methods used is below 40°C and is suitable for drying herbal medicine raw materials because it can protect the active compounds from damage. Drying natural materials at high temperatures for a long time can damage the chemical components contained in the materials. Statistically, there were no significant differences in temperature and humidity among the different drying methods used for drying the tubers and leaves of rat taro. The drying method did not significantly affect temperature or humidity (*p* > 0.05). The drying of rat taro tubers can be performed using direct sunlight, sunlight filtered through black fabric, or an oven, with careful attention to temperature and humidity conditions. The drying conditions for tubers are a temperature range of 37.5°C–39.0°C, RH of 45.0%–54.5%, and a drying duration of approximately 10–16 h.

**Table 1:** Effect of drying methods on temperature, humidity, drying time, and dry weight yield of rat taro tubers and leaves.

**Drying methods**	**Observation result**							
	**Temperature**		**Humidity**		**Drying time**		**Yield**	
	**Tubers**	**Leaves**	**Tubers**	**Leaves**	**Tubers**	**Leaves**	**Tubers**	**Leaves**
Sun drying	37.5a	32.5a	54.5a	52.5a	15.0 ± 3.0a	21.0 ± 3.0a	33.32a	8.87a
Modified sun drying	39.0a	36.0a	45.0a	46.5a	10.0 ± 3.0a	26.0 ± 2.0a	29.32a	9.28a
Oven drying	38.0a	37.0a	48.0a	51.5a	16.0 ± 2.0a	20.0 ± 2.0a	22.49a	8.75a

*Note:* Numbers followed by the same letters in the same column are not significantly different at 5% of DMRT.

### 3.3. Drying Time

The drying time for rat taro in the three different drying methods was not significantly different. Table 1 shows the effect of drying methods on temperature, humidity, drying time, and dry weight yield of rat taro tubers and leaves. The drying temperature, humidity, and water content of the material will affect the speed of the drying process. The results indicate that the modified sun drying method provides the shortest drying time of 10 h for producing rat taro tuber simplicia. The use of fabric covers influences the drying time of tuber products during sunlight exposure compared to uncovered products. The use of fabric covers significantly influences the drying time of tuber products during sunlight exposure by affecting humidity and temperature levels. Covered products tend to dry faster due to increased temperatures and reduced humidity, which are critical factors in the drying process. Previous research has demonstrated that covered solar dryers are able to reduce drying duration considerably, with some products requiring 20–44 h less than conventional sun drying methods [20].

As shown in Table 1, higher drying temperatures resulted in shorter drying times for both tubers and leaves. Increasing the drying temperature enhances moisture removal and consequently reduces drying time [21]. The drying process of tubers is faster than leaves, as tubers contain less water compared to leaves. In addition to moisture content, the type of material and the composition of the dried material affect the moisture diffusion. Effective moisture diffusivity is anticipated to be the only physical mechanism for the transfer of water from the core to the material surface during the drying process [15]. The present research is comparable to a previous study that employed an oven dryer. A previous study reported that drying rat taro tubers at 40°C, 50°C, and 60°C required 10.0–10.5, 6.5–7.0, and 4.5–5.0 h, respectively, to achieve a moisture content below 10%. For rat taro leaves dried at 60°C, the moisture content decreased from 90% to 92% to less than 10% (wet basis) after 10.44 h of drying [18]. Statistical analysis indicated that variations in drying time across all drying methods applied to rat taro tubers did not result in significant differences in dry weight yield (*p* > 0.05).

### 3.4. Simplicia Yield

**Figure 3:**
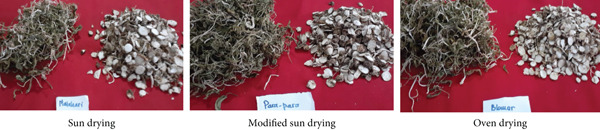
Rat taro simplicia from various types of drying.

The results showed that various drying methods obtained a dry weight yield of rat taro tubers from 22.49% to 33.32%. The percentage yield of simplicia between the sun dryer and the modified sun dryer covered with a black fabric was not significantly different. The percentage yield of simplicia was between 29.32% and 33.32%. Meanwhile, the yield of leaf simplicia ranges from 8.75% to 9.28%. The drying method had a significant effect on the yield of rat taro tuber simplicia, while the yield of leaves was not significantly different between drying methods. Figure 3 shows rat taro simplicia from various types of drying methods. Statistically, although different drying methods resulted in variations in temperature, humidity, and drying duration, they did not significantly affect the dry weight yield of rat taro tubers (*p* > 0.05). The contribution of temperature, humidity, and drying time to the tuber yield was 0.88%, 0.62%, and 0.81%, respectively. Tuber drying can be carried out using direct sunlight, sunlight filtered with a black cloth, or an oven, with careful consideration of temperature and humidity.

In contrast, for the leaves, the drying method significantly affected the dry weight yield. Modified sun drying produced a higher dry weight yield compared to other methods. The significance value of the drying method was 0.039 (< 0.05), indicating a statistically significant effect on the dry weight yield of the leaves. However, post hoc analysis revealed no significant differences in dry weight yield among the different drying methods. Drying leaves under direct sunlight is considered economically feasible when environmental conditions are maintained at a temperature of 32.5°C–37.0°C, RH of 46.5%–52.5%, and a drying duration of approximately 21 h.

### 3.5. Moisture Content

The moisture content of tuber simplicia ranges from 8.75% to 10.62% and leaves 8.27% to 10.84%. Table 2 shows the quality characteristics of rat taro simplicia from various methods of drying. The higher the moisture content of simplicia, the higher the dry weight yield. The oven dryer produced the lowest moisture content of the tuber. The highest moisture content of the tuber was from the sun drying method. In statistical analysis, the tuber moisture content between various drying methods was not significantly different. The moisture content of rat taro tubers and leaves dried using modified sun drying and oven drying methods met the Indonesian Materia Medika (MMI) standard for simplicia, with a maximum of 10% moisture content for herbal medicine raw material. The water content of tuber and leaf simplicia ranges from 8.27% to 9.63%.

**Table 2:** The effect of three drying methods on the quality characteristics of rat taro simplicia.

**Drying methods**	**Observation result**							
	**Moisture content (%)**		**Ash content (%)**		**Water-soluble extractive content (%)**		**Alcohol-soluble extractive content (%)**	
	**Tuber**	**Leaf**	**Tuber**	**Leaf**	**Tuber**	**Leaf**	**Tuber**	**Leaf**
Sun drying	10.84a	10.62a	4.00a	15.11a	11.07a	35.00a	2.28a	6.35b
Modified sun drying	8.27a	9.63a	4.22a	15.10a	12.81a	37.05a	2.95a	8.71a
Oven drying	9.46a	8.75a	4.28a	14.61a	10.92a	39.10a	1.88a	9.19a

*Note:* Numbers followed by the same letters in the same column are not significantly different at 5% of DMRT.

### 3.6. Ash Content

The total ash content of rat taro tuber simplicia ranged from 4% to 4.28%, while the leaves ranged from 14.61% to 15.11%. The total ash content in leaves is higher than in tubers. The ash content in plant material refers to the total amount of minerals present in the plant materials [22]. The ash content indicates the presence of mineral elements in the tubers and leaves of rat taro. The drying method has no significant effect on the total ash content in tuber and leaf simplicia. Ash content can form in fresh material during the drying process, and physical and chemical changes occur in the material. No further analysis was conducted to identify the specific mineral constituents. Instead, the determination of total ash content was intended to serve as a reference for comparison with the simplicia standards established in the Indonesian Pharmacopoeia. The higher the total ash content of simplicia, the higher the mineral element content. Ash content varied depending on the drying method used, which was influenced by drying temperature and moisture content [23].

### 3.7. Water‐Soluble and Alcohol‐Soluble Extract Content

The result showed the rat taro tuber simplicia contain water‐soluble extract content ranging from 10.92% to 12.81% and alcohol‐soluble extract content from 1.88% to 2.95%. The water‐soluble extract content in leaf simplicia was 35%–39.10%, and the alcohol‐soluble extract content was 6.35%–9.19%. Rat taro simplicia contains a higher level of water‐soluble extracts than alcohol‐soluble extracts. Leaves have higher levels of water‐soluble extract and alcohol‐soluble extract than the tuber. Water is an efficient solvent for extracting polar molecules, whereas less polar compounds are more effectively extracted using organic solvents. Water might enhance the efficiency of the extraction process by helping the diffusion of extractable components like polyphenols through plant tissues [24]. The phytochemical screening test of the rat taro leaf ethanol extract indicated that the compound consisted of flavonoid, steroid, and tannin [25]. Rat taro simplicia contains higher levels of water‐soluble than alcohol‐soluble contents. Observations of water‐soluble content and alcohol‐soluble content aim to illustrate the chemical processes involved in hydration and the molecular dynamics of ethanol–water mixtures, enhancing our understanding of their properties and behaviors in solutions [26].

The polarity of the solvent significantly affects extraction yield; highly polar solvents yield more extract but contain lower phenolic and flavonoid content compared to nonpolar solvents [27]. The drying method has a significant effect on the water‐soluble and alcohol‐soluble extract content of rat taro simplicia. The water‐ and alcohol‐soluble content of the leaves with the oven drying and modified sun drying methods is higher than the sun drying method. Rat taro can be extracted using water as a solvent or a combination of water and ethanol.

### 3.8. Flavonoid

The average flavonoid content of rat taro tuber simplicia ranged from 0.2 to 0.3 mg QE/g, whereas that of the leaves ranged from 4.1 to 5.6 mg QE/g (Table 3). The flavonoid content of leaves simplicia were higher than the tubers. Table 3 shows the flavonoid content of tuber and leaf simplicia from various drying methods. The drying method has a significant effect on the flavonoid content of rat taro simplicia. The oven drying method produced the highest flavonoid content. The average temperature of the rat taro drying method ranges from 36°C to 39°C. The rate of heating significantly affects flavonoid degradation during drying. Higher temperatures accelerate oxidation, leading to reduced flavonoid efficacy [28]. The drying method influences the degradation of plant simplicia bioactive compounds [29, 30]. Rat taro leaves have higher levels of flavonoids than the tubers. This is likely attributable to the greater diversity and abundance of flavonoid compounds present in the leaves compared to the tubers.

**Table 3:** Flavonoid content in rat taro simplicia from various drying methods.

**Drying methods**	**Flavonoid content (mg QE/g)**	
	**Tuber**	**Leaf**
Sun drying	0.3a	4.1c
Modified sun drying	0.2b	4.9b
Oven drying	0.3a	5.6a

*Note:* Numbers followed by the same letters in the same column are not significantly different at 5% of DMRT.

Within total flavonoid content in rat taro leaves of 4.1–5.6 mg QE/g (Table 3), many researches have been extensively conducted to identify the flavonoid compounds extracted using organic solvents. The previous study reported that flavonoids from the ethyl acetate fraction of the methanol extract of TFB leaves were isolated and identified as 6‐C‐glucosyl apigenin, namely, isovitexin (apigenin‐6‐C‐glucoside) [31]. Meanwhile, profiling of the ethanolic leaf extract by UHPLC‐Q‐Orbitrap HRMS identified 15 phenolic compounds, namely, flavones: apigenin C‐hexoside‐C‐pentoside and vitexin (apigenin 8‐C‐glucoside); flavonols: kaempferol 3‐O‐rutinoside, kaempferol, and kaempferol 3‐O‐(6″‐acetyl‐galactoside)‐7‐O‐rhamnoside; hydroxybenzaldehyde: 4‐hydroxybenzaldehyde and p‐anisaldehyde; hydroxybenzoic acids: p‐coumaric acid, quinic acid, cinnamic acid, ferulic acid, and p‐coumaric *α*‐glucoside acid; hydroxy propanoic acids: caffeic acid and caffeic O‐glucoside acid; and aldehyde phenolics of vanillin [32]. The phytochemical compounds contained in the rat taro tuber are methyl esters of hexadecanoic acid, octadecanoic acid, 9‐octadecenoic acid, and 9,12‐octadecadienoic acid [33].

Isovitexin, as a flavonoid, has been identified in the leaves of rat taro [34]. Another flavonoid compound is flavonols, such as kaempferol 3‐O‐rutinoside, kaempferol, and kaempferol 3‐O‐(6″‐acetyl‐galactoside)‐7‐O‐rhamnoside, and flavones, such as apigenin C‐hexoside‐C‐pentoside and vitexin (apigenin 8‐C‐glucoside). The other phytochemical compounds detected in the leaves are hydroxybenzaldehyde, 4‐hydroxybenzaldehyde, p‐anisaldehyde, p‐coumaric acid, quinic acid, cinnamic acid, ferulic acid, p‐coumaric *α*‐glucoside acid, caffeic acid, caffeic O‐glucoside acid, and vanillin [32]. Meanwhile, the phytochemical compounds contained in the tuber are methyl esters of hexadecanoic acid, octadecanoic acid, 9‐octadecenoic acid, and 9,12‐octadecadienoic acid [33].

Statistical analysis showed that the flavonoid content of both tubers and leaves did not differ significantly between the drying methods. The drying method has a statistically significant effect on the flavonoid levels of rat taro (*p* = 0.009). Rat taro leaves contained higher levels of flavonoids compared to the tubers. Accordingly, the flavonoid content in the leaves was significantly higher than that in the tubers.

### 3.9. Antioxidant Activities

The IC_50_ value of rat taro tuber simplicia ranges from 6477.70 to 8847.77 ppm, while that of the leaves ranges from 585.01 to 1189.63 ppm. Rat taro plant has been found to possess antioxidant potential due to its high content of total phenolic compounds [35]. The IC_50_ value of leaves is smaller than tubers. It was revealed that rat taro leaves have higher antioxidant activity than tubers. The smaller IC_50_ value indicates that the antioxidant activity is stronger. The IC_50_ value of leaves is smaller than tubers. It was revealed that rat taro leaves have higher antioxidant activity than tubers. The smaller IC_50_ value indicates that the antioxidant activity is stronger. To achieve 50% free radical scavenging activity, 585.01–634.44 ppm of rat taro leaf simplicia extract is required, whereas 6477.70–7989.17 ppm is needed when using tuber extract. Rat taro plant has been found to possess antioxidant potential due to its high content of total phenolic compounds [35]. In addition, flavonoid compounds also contribute to antioxidant activity. A previous study showed that rat taro leaves contain the flavonoid isovitexin [34]. Rat taro has also been identified as containing several compounds classified as flavonols, namely, kaempferol 3‐O‐rutinoside, kaempferol, and kaempferol 3‐O‐(6″‐acetyl‐galactoside)‐7‐O‐rhamnoside [32]. Kaempferol has been proven to have antioxidant activity [36]. Based on the analysis of flavonoid content, it is known that the flavonoid levels in rat taro leaves are higher than in the tuber.

The oven drying method produced tuber and leaf simplicia with the highest antioxidant activity compared to the sun drying and modified sun drying methods. The drying method influenced antioxidant activity (*p* = 0.028). The drying method significantly influences the concentration of secondary metabolites, including phenolic compounds, and the biological activity of plants postharvest and throughout storage [37]. These changes are influenced by the duration of the drying process as well as environmental factors, including temperature and RH [38]. The oven drying method certainly has different temperatures and process conditions compared to sun drying. Prolonged drying durations frequently result in the oxidation of phenolic components, diminishing antioxidant capacity and anthocyanin levels [39]. The antioxidant activity of tuber simplicia did not differ significantly among the various drying methods. Similarly, no significant differences were observed in the antioxidant activity of leaf simplicia across the different drying methods. Table 4 shows the IC_50_ values of tuber and leaf simplicia of rat taro from various drying methods.

**Table 4:** IC_50_ values of tuber and leaf simplicia of rat taro from various drying methods.

**Drying methods**	**I** **C**_50_ ** value (ppm)**	
	**Tuber**	**Leaf**
Sun drying	8847.77a	1189.63a
Modified sun drying	7989.17ab	634.44b
Oven drying	6477.70b	585.01b

*Note:* Numbers followed by the same letters in the same column are not significantly different at 5% of DMRT.

The inhibition percentage of the ethanolic extract is shown in Table 5. The inhibition at the concentration of 250 ppm ranged around 12.80*%* ± 3.44*%*–24.17*%* ± 3.31*%*. The result is comparable to a previous study that the free radical scavenging activity of ethanol extract of rat taro at a concentration of 250 *μ*g/mL has an inhibition value of 35.06*%* ± 3.05*%* [32]. Meanwhile, the ethyl acetate and dichloromethane extracts of rat taro showed higher inhibition, in which at a concentration of 100 *μ*g/mL exhibit 77.6*%* ± 0.9*%* and 70.5*%* ± 1.7*%* [40]. Drying temperature affects the stability of antioxidant activity [41]. Higher drying temperatures decrease antioxidant activity, as demonstrated by reduced DPPH radical scavenging activity and ferric ion–reducing antioxidant power (FRAP) [42]. Antioxidant activity is related to the bioactive compounds contained in the ingredients. The high antioxidative activity of the ethanolic extract could be attributed to the kaempferol (flavonoids) content. Flavonoid compounds have been scientifically proven to have antioxidant activity [36]. The oven drying method produced rat taro leaf simplicia with higher antioxidant activity compared to the sun drying and modified sun drying methods. The drying process in the oven dryer has a more stable temperature than the sun dryer. Sun drying has a fluctuating temperature because it depends on the weather. The relatively lower quantities of the proximate content in the fruits dried under the sun drying and modified sun drying methods could be due to the increased temperatures and reduced drying times, which have probably accelerated the fruit drying process [43]. Duncan′s post hoc test revealed that the optimal treatment was obtained using either the modified sun drying or oven drying method, combined with an inhibitory concentration of 2000 ppm, which resulted in an inhibition rate of 89.81%–90.76%.

**Table 5:** Percentage of inhibition of ethanol extract of rat taro leaves.

**Drying methods**	**Inhibition percentage**				
	**Concentration of rat taro ethanol extract (%)**				
	**125** **ppm**	**250** **ppm**	**500** **ppm**	**1000** **ppm**	**2000** **ppm**
Sun drying	6.16 ± 1.88	12.80 ± 3.44	23.93 ± 6.53	46.21 ± 10.11	79.86 ± 3.49
Modified sun drying	11.14 ± 1.28	21.56 ± 3.42	41.94 ± 6.43	76.30 ± 9.10	90.76 ± 4.40
Oven drying	12.32 ± 1.03	24.17 ± 3.31	44.79 ± 5.2	81.99 ± 11.10	89.81 ± 3.39

*Note:* Numbers followed by the same letters in the same column are not significantly different at 5% of DMRT.

## 4. Conclusion

The drying method affects the drying time, antioxidant activity, and flavonoid content of rat taro simplicia. The water‐ and alcohol‐soluble content, antioxidant activity, and flavonoid content of rat taro leaf simplicia are higher than the tubers. The drying method has a significant effect on the quality of simplicia. The oven drying method at a temperature of 37°C–38°C produces high‐quality rat taro simplicia. Rat taro tubers can be dried using direct sunlight, modified sun drying (covered with black fabric), or an oven, with careful control of temperature and humidity conditions. The drying conditions for tubers are a temperature range of 37.5°C–39.0°C, RH of 45.0%–54.5%, and a drying duration of approximately 10–16 h. For the leaves, direct sunlight drying is considered cost‐effective when environmental conditions are maintained within a temperature range of 32.5°C–37.0°C, RH of 46.5%–52.5%, and a drying duration of approximately 21 h. The use of modified sun drying or oven drying, combined with an inhibitory concentration of 2000 ppm, resulted in optimal inhibition levels ranging from 89.81*%* ± 3.39*%* to 90.76*%* ± 4.40*%*. However, the findings are limited to physicochemical parameters, flavonoid levels, and antioxidant activity. Other important attributes for raw material quality were not assessed. Future research should address these aspects and employ advanced analytical techniques to provide a more comprehensive understanding, thereby supporting small‐scale industries in producing raw materials with consistent quantity, quality, and continuity to meet the standards of herbal medicines.

## Conflicts of Interest

The authors declare no conflicts of interest.

## Funding

No funding was received for this manuscript.

## Data Availability

The data used to support the findings of this study can be obtained from the corresponding author upon reasonable request.
